# Staged Reconstruction of a Complex Zone 3 Injury Involving Neurovascular and Tendon Repair

**DOI:** 10.7759/cureus.90803

**Published:** 2025-08-23

**Authors:** Aulon Jerliu, Herbert Downton Ramos, Jacob I Jabbour

**Affiliations:** 1 Surgery, University of Connecticut School of Medicine, Farmington, USA; 2 General Surgery, University of Connecticut School of Medicine, Farmington, USA; 3 General Surgery, Larkin Community Hospital, Miami, USA

**Keywords:** hand trauma surgery, microsurgical repair, nerve reconstruction, tendon laceration, zone 3 hand injury

## Abstract

We present a case of a 67-year-old male who sustained a complex glass injury to the right palm involving Zone 3. Intraoperative exploration revealed full-thickness lacerations of the flexor digitorum profundus (FDP) and flexor digitorum superficialis (FDS) tendons to all four fingers, a 22 mm median nerve gap requiring cabled nerve allograft repair, laceration of the deep motor branch of the ulnar nerve and fourth common digital nerve requiring conduit-assisted repairs, and a segmental laceration of the ulnar artery requiring microsurgical reconstruction. The patient underwent staged reconstruction over two operations, including tendon repairs, nerve grafting with cabled decellularized frozen nerve allograft, vascular repair, and soft tissue coverage with adjacent tissue transfer. This case highlights the complexity of Zone 3 hand injuries and underscores the importance of staged surgical decision-making, nerve reconstruction strategies, and prioritization of neurovascular and tendon repair in high-impact penetrating glass trauma. The patient provided written informed consent for publication of all clinical details and images.

## Introduction

Complex lacerations of the palm present a significant challenge to hand surgeons due to the compact arrangement of vital neurovascular and musculoskeletal structures. Zone 3, defined as the region distal to the transverse carpal ligament but proximal to the digital tendon sheaths, is a conduit for nearly all flexor tendon and sensory nerve pathways of the digits. While the primary motor branches to the thenar, hypothenar, and interosseous muscles usually arise more proximally (Zones 4-5 or distal forearm), distal extensions of these branches may still be encountered in Zone 3 and placed at risk during high-impact lacerations. Injuries in this region often involve multiple structures, including flexor tendons, digital nerves, and palmar vascular arches, necessitating meticulous planning and reconstruction strategies [1,2].

The management of combined injuries to the median and ulnar nerves, flexor tendons, and arterial systems has been described in isolated cases, but the staged approach for simultaneous reconstruction remains underreported. Staging may be necessary not only due to systemic comorbidities, but also when intraoperative findings reveal severely contused nerve tissue, patient instability under anesthesia, or anticipated operative times that exceed safe limits. These considerations informed our decision-making in the present case. Nerve allografts and conduits have expanded treatment options, especially in segmental nerve loss, minimizing donor site morbidity [3,4]. Similarly, advanced tendon repair techniques like multi-strand core suturing have improved outcomes [5,6].

## Case presentation

**Figure 1:**
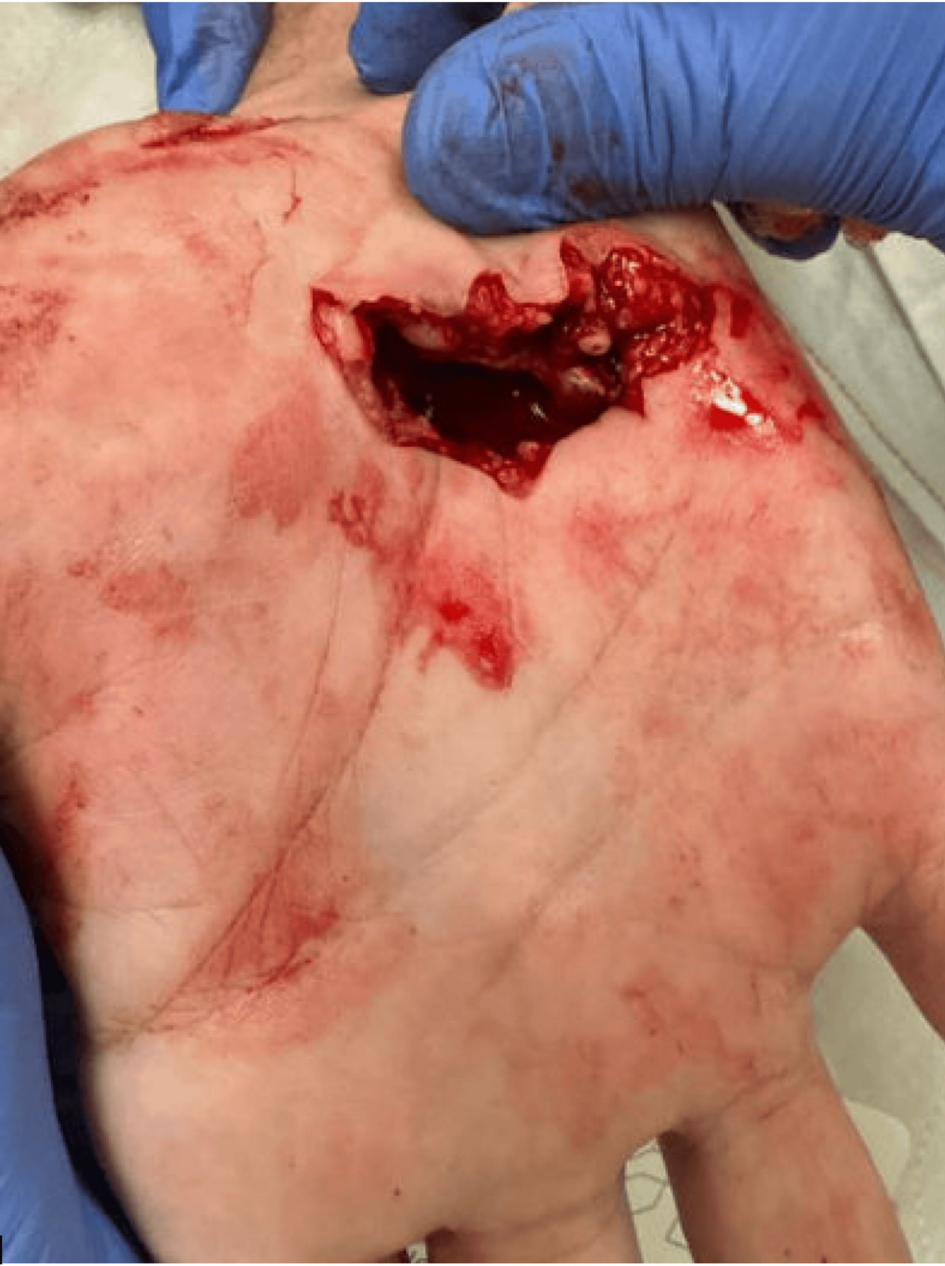
Photo of the initial laceration seen in the trauma bay. Multiple structures can be seen transected.

A 67-year-old right-hand dominant male with a history of alcohol dependence and hypertension presented after sustaining a deep glass laceration to his right palm (Figure 1). On arrival to the trauma bay, he had heavy bleeding, severe palmar pain, inability to actively flex his fingers, and decreased perfusion of the ulnar digits. Examination revealed a 6 cm open transverse laceration distal to the carpal tunnel with exposed tendons and suspected neurovascular injury. The trauma team ligated the bleeding ulnar artery, and the patient was stabilized for emergent operative exploration.

The ulnar artery had been temporarily ligated by the trauma team prior to surgical consultation.

First operation

**Figure 2:**
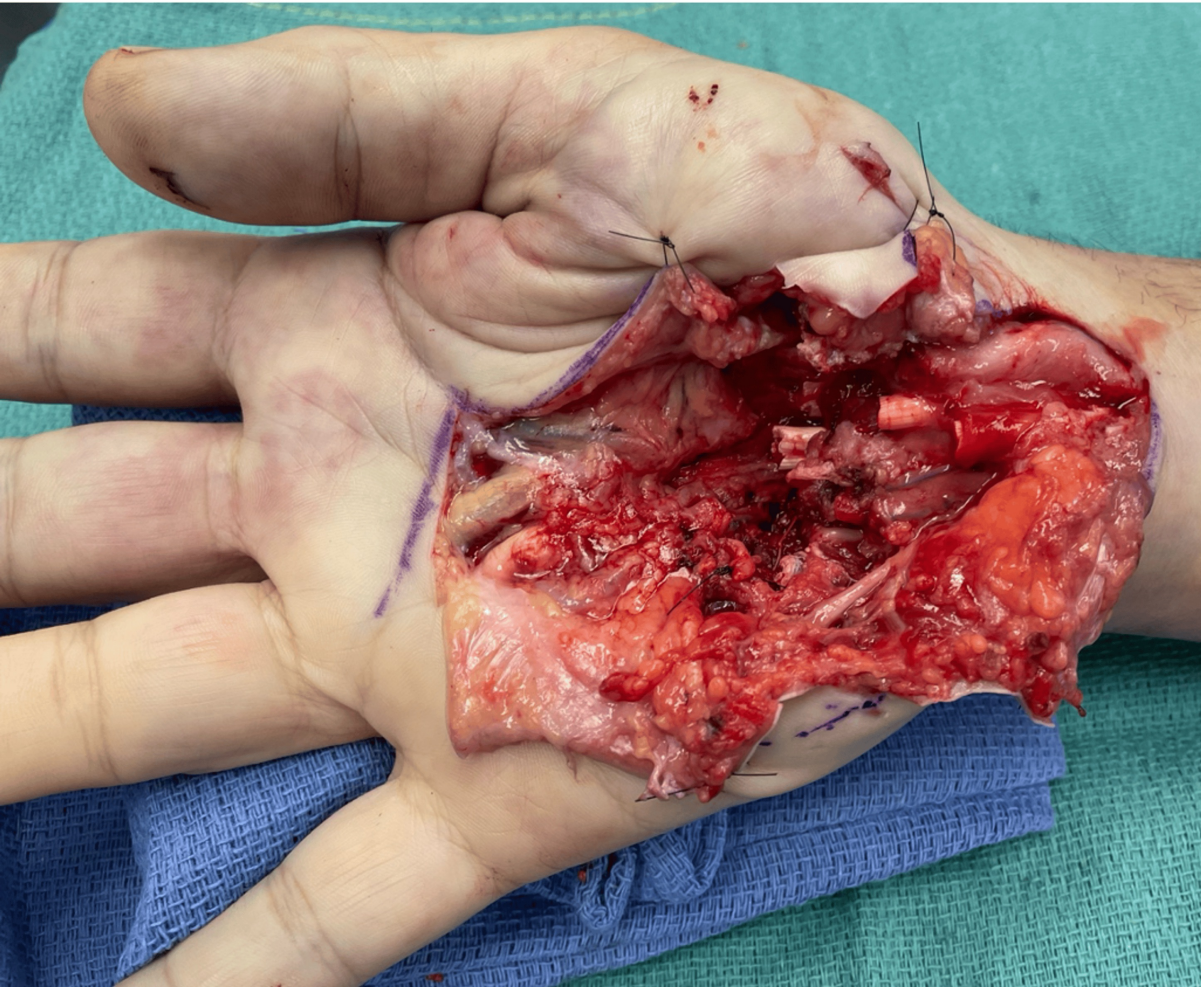
Photo of intra-operative evaluation of the hand after surgical exposure showing full-thickness transections of FDP and FDS to all fingers, a transected median nerve, lacerations to the deep motor branch of the ulnar nerve and fourth common digital nerve, segmental injury to the ulnar artery. FDP: flexor digitorum profundus; FDS: flexor digitorum superficialis (FDS).

A T-shaped wound extension provided adequate exposure. Intraoperative findings included full-thickness transections of flexor digitorum profundus (FDP) and flexor digitorum superficialis (FDS) to all fingers, a transected and contused median nerve, lacerations to the deep motor branch of the ulnar nerve and fourth common digital nerve, segmental injury to the ulnar artery, and muscle injuries (Figure 2).

Ulnar artery reconstruction utilized microsurgical end-to-end repair. Microsurgical repairs of ulnar nerve branches were performed via conduit-assisted repair with porcine intestinal submucosa nerve connectors. Muscle injuries were repaired with absorbable polyfilament. Given extensive injuries and the patient’s comorbidities and deteriorating status, it was decided to forego definitive repair of additional structures. Flexor tendons and the median nerve were tagged for staged repairs.

Second operation

**Figure 3:**
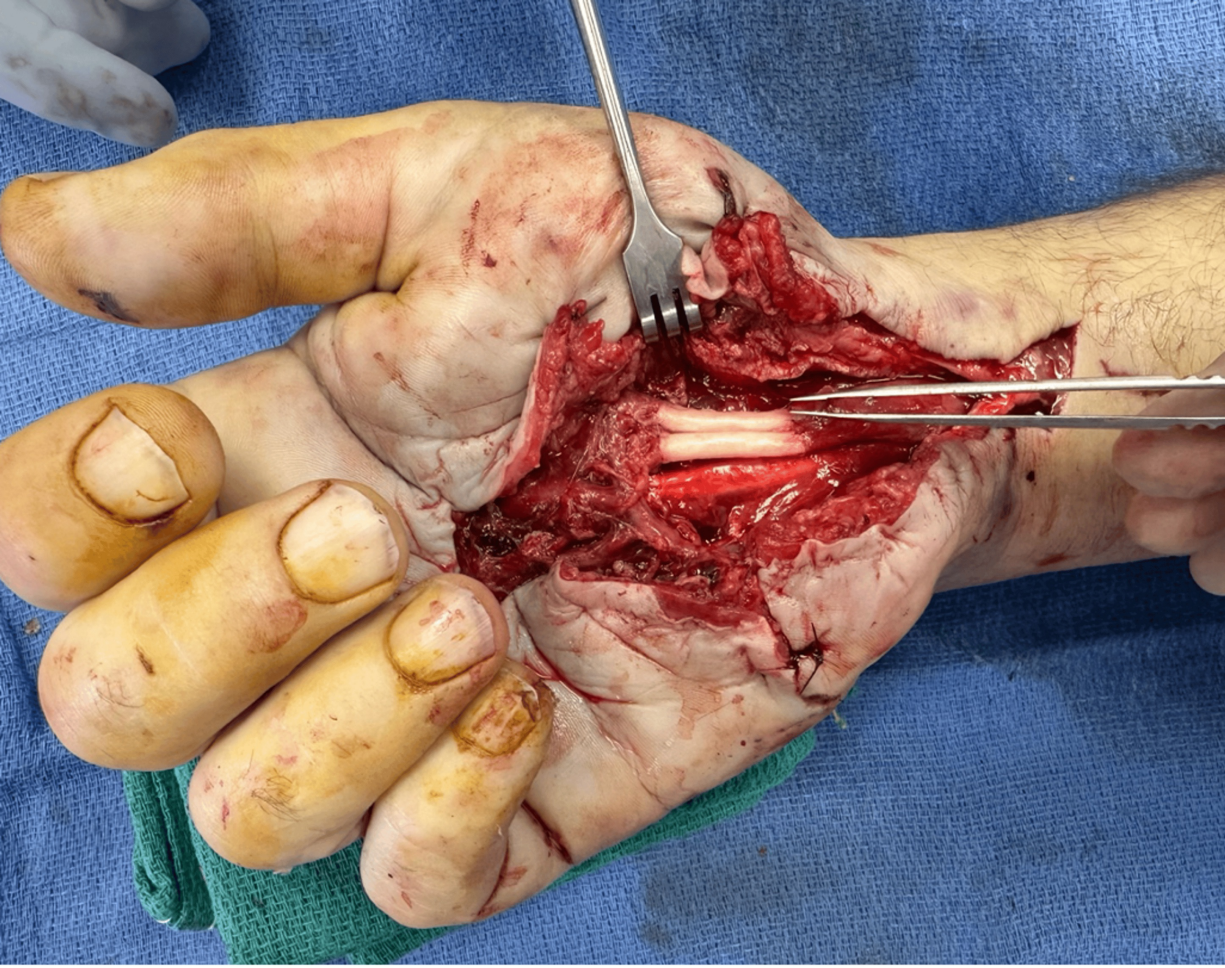
Photo of the median nerve cabled allograft repair superficial to the already repaired flexor tendons.

Two days later, delayed median nerve and tendon repairs were performed. The median nerve was noted to have considerable contusion at the index operation. After the short interval, the devitalized portion of the nerve was more definitively able to be declared and resected to healthy margins. The median nerve was reconstructed in a tension-free manner, using two cabled decellularized frozen nerve allografts bridging a 22 mm gap with a 9 mm diameter (Figure 3). Tendons were repaired using an 8-core modified Kessler technique augmented with synthetic polyfilament sutures.

The third palmar interosseous and lumbricals to the index, middle, and ring fingers were repaired using 3-0 Vicryl (Ethicon, Raritan, NJ, USA) figure-of-eight epimysial sutures placed 2 mm from the muscle edge, with the hand held in approximately 45° metacarpophalangeal (MCP) flexion and slight IP extension. A larger caliber was selected to ensure adequate tensile strength and resistance to pull-out in the context of a high-energy wound bed. Each repair was left with about 5 mm slack to allow excursion. These repairs overlay the FDP tendon repairs and provide a biologic gliding surface to support tendon function. An adjacent tissue transfer (12 × 4 cm), created from an L-shaped, undermined wound, was designed as a pedicled palmar V-Y rotation-advancement flap based on distal common-palmar perforators. A dorsal blocking splint was applied.

Outcome

In the immediate post-operative period, perfusion to all digits remained adequate, and no acute complications were observed. Initiation of supervised hand therapy was delayed until postoperative day 10 due to the patient’s episode of active alcohol withdrawal, which was consistent with his history of chronic alcohol dependence and necessitated ICU monitoring. Despite this setback, the patient has demonstrated encouraging early clinical signs.

Two months postoperatively, perfusion to the hand remains robust, with improving sensation to sharp pinprick, indicating promising evidence of nerve recovery across the grafted repair site. There is active isolated flexion at each proximal interphalangeal (PIP) and distal interphalangeal (DIP) joint expressed. He continues to work through the active range of motion therapy. Due to his delayed initiation with the active range of motion (ROM) protocol, there is some expected difficulty in tendon excursion and persistent stiffness at the digits.

At four months postoperative, the patient reported no pain, has returned to work handling tools without fatigue, and demonstrates encouraging early nerve and tendon recovery with improving sharp pinprick discrimination, active PIP flexion to 110°, isolated DIP motion, active MCP flexion ranging from 70-90° with simultaneous IP extension enabling an intrinsic-plus posture, total active motion exceeding 200° in the index, middle, and ring fingers without clawing or swan-necking, fingertip reach within 1 cm of the distal palmar crease, and functional opposition of the thumb to the index and long fingers with key pinch to a domino, while therapy focused on persistent extrinsic flexor tightness and limited composite fist formation.

## Discussion

This case highlights the utility of a staged surgical approach in managing Zone 3 palmar trauma with multi-structure injury. Initial priorities in complex hand injuries include revascularization, infection control, and stabilization of the wound environment. In this case, infection control was achieved by thorough irrigation, sharp debridement of nonviable tissue, staged reconstruction to shorten initial operative time, soft tissue coverage with a V-Y adjacent tissue transfer, and perioperative intravenous antibiotics. Together, these steps minimized the risk of infection and optimized the wound bed for delayed definitive repair. The decision to stage tendon and median nerve repair in favor of immediate vascular and ulnar nerve branch reconstruction reflects current principles in complex limb salvage and damage control hand surgery [7,8]. In this case, staging was chosen based on three factors: (1) pronounced contusion of the median nerve that precluded immediate definition of healthy fascicles; (2) increased intraoperative instability; and (3) an estimated additional two to three hours to complete the procedure. These criteria met accepted thresholds for elective damage-control hand surgery. Like other damage control principles, patient resuscitation and addressing the most pressing surgical injury must be prioritized. Additionally, surgical wound/contamination, hospital resources, availability of a microsurgical team or hand surgeon, appropriate supplies, and other ancillary details can affect a surgeon’s decision in performing definitive repairs at the index surgery versus staging the repair.

Staged reconstruction of the median nerve using cabled decellularized frozen nerve allografts allowed for re-establishment of continuity in the setting of a large defect while minimizing donor site morbidity. Nerve allografts have demonstrated comparable outcomes to autografts in digital nerve repair when gaps are less than 3 cm and the wound bed is optimized [3,4]. Additionally, conduit-assisted repairs for distal nerve branches such as the deep motor branch of the ulnar nerve and the fourth common digital nerve provide tension-free coaptation and protect regenerating axons from scar invasion, with good functional outcomes [8,9]. Timely nerve repair is essential for optimal functional recovery. Immediate repair within 24 hours is ideal; however, if immediate repair isn't feasible, performing the repair within 14 days is recommended to optimize axonal regeneration and minimize fibrosis.

Multistrand tendon repairs using 6-8 core suture techniques improve the strength of flexor tendon constructs, facilitating early active mobilization protocols, which are crucial in minimizing adhesions and joint stiffness [5,6]. Flexor tendon repair is ideally performed within the first seven days post-injury to reduce the risk of adhesion formation. Repair of the lumbricals and interossei, although not always emphasized in trauma cases, plays an important role in restoring fine motor coordination and intrinsic balance of the hand [10]. Local tissue rearrangement through adjacent tissue transfer allowed for robust and tension-free coverage of neurovascular repairs, thereby reducing the risk of exposure, desiccation, or secondary breakdown, which are key factors in successful tendon and nerve regeneration [7].

To our knowledge, this is the first report to document three elements: (i) tension-free cabled allograft reconstruction of a > 2 cm median nerve gap in Zone 3, (ii) deliberate staging of tendon and median nerve repair under damage-control-hand-surgery principles, and (iii) immediate sensate V-Y flap coverage of simultaneous vascular, nerve, and tendon repairs. The application of cabled allografts for a large median nerve gap and multi-point nerve conduit repairs provides practical guidance for managing extensive segmental injuries. Beyond technical reconstruction, this case illustrates how a damage control strategy can be adapted to the hand, offering meaningful recovery despite delayed rehabilitation.

## Conclusions

High-impact penetrating glass injury Zone 3 injuries require staged and structured approaches. Nerve conduits, allografts, multistrand tendon repairs, and meticulous soft tissue coverage significantly enhance functional recovery. This case highlights the complexity of Zone 3 hand injuries and underscores the importance of staged surgical decision-making, nerve reconstruction strategies, and prioritization of neurovascular and tendon repair in high-impact penetrating glass penetrating trauma.
